# *IDH1* Mutation in Gliomas in Mosul City - Iraq

**DOI:** 10.3889/oamjms.2015.041

**Published:** 2015-04-29

**Authors:** Mohammed Sami Saeed

**Affiliations:** Mosul Medical College, University of Mosul, Dept. of Pathology, Mosul Medical College Al-Shifaa Quarter, Mosul, Ninevah 41002, Iraq

**Keywords:** *IDH1*, gliomas, immunohistochemical, adult, pediatric

## Abstract

**BACKGROUND:**

*IDH1* (isocitrate dehydrogenase 1) mutation might be encounter in the low grade glioma and directs the progression of the tumor to a higher grade.

**OBJECTIVE:**

To assess the frequency of *IDH1* mutations in gliomas and to correlate the *IDH1* positivity with the type and grade of tumors, the age and sex of the patients.

**MATERIAL AND METHODS:**

A retro– and prospective case series study. One hundred and nine cases of intracranial gliomas were collected between 2008 and 2014 from Mosul Private Laboratories and Al-Jamboree Teaching Hospitals in Mosul. *IDH1* mutations were assessed immunohistochemically using anti-*IDH1* R132H mouse monoclonal antibody.

**RESULTS:**

*IDH1* mutation was perceived in 34.86% of gliomas. In adult gliomas, the secondary glioblastoma and the low-grade astrocytoma had the greatest values of *IDH1* positivity (88.88% and 62.5% respectively), followed by oligoastrocytoma/oligodendroglioma (50.0%), and anaplastic astrocytoma (47.36%). The primary glioblastomsa showed 17.64% *IDH1* positivity. Males and females expressed the *IDH1* equally. While, there was no role of *IDH1* in pediatric gliomas.

**CONCLUSION:**

*IDH1* mutation is commonly present in adult gliomas particularly in low-grade gliomas, and secondary glioblastoma, with equal sex distribution, but it has no role in pediatric gliomas.

## Introduction

Glial tumors (or gliomas) account for 40%–45% of all primary intracranial tumors. Therefore, they are considered the most common type of primary brain tumors. Gliomas are classified as grade I to grade IV according to histopathological and clinical criteria established by the WHO [1]. This group of tumors includes specific histologic subtypes, the most common of which are astrocytomas, oligodendrogliomas, and ependymomas [1]. WHO grade I gliomas, has an indolent growth, often considered to be benign, and rarely, if ever, evolve into higher-grade lesions. By contrast, gliomas of WHO grade II or III are aggressive tumors, usually invasive, progress to higher-grade lesions, and have a poor outcome [1].

Progression of glioma to a higher grade tumor is multistep process involving many genes and characterized by genetic alterations and mutation accumulation, these include TP53, PTEN, CDKN2A, and EGFR [2]. Recent studies suggested that mutations in the gene encoding for cytosolic NADP+ dependant *IDH1* (isocitrate dehydrogenase 1) might occur after the formation of a low-grade glioma and direct the progression of the tumor to a glioblastoma [3, 4].

*IDH1* is a member of IDH gene family, located on chromosome 2q33.3 and encodes for the cytosolic NADP+ dependant isocitrate dehydrogenase enzyme. The product protein catalyze the cytosolic oxidative decarboxylation of isocitrate to alpha-ketoglutarate, and resulting in the production of reduced form of NADP+ (NADPH) which is play an important role in the cellular control of oxidative damage [5-7]. Gene mutation alters the enzymatic property of *IDH1* and leads to increase conversion of alpha-ketogluterate to 2-hydroxyglutarate (2HG) metabolite and decreased production of NADPH, and accordingly reduced glutathione. These alterations may raise the oxidative stress level in mutant *IDH1* cells and acting as an oncogen [8-10].

*IDH1* mutation has been observed as an early evidence and in high frequency (50%-93%) among astrocytomas, oligodendrogliomas, oligodendro-gliomas and secondary glioblastomas, while rarely occurs in primary glioblastoma [2-6,11,12].

Mutant *IDH1* anaplastic astrocytomas, glioblastomas and oligodendroglial tumors have independent favorable prognostic factor particularly for grade III gliomas, and usually associated with increased progression-free survival and overall survival and may exceed other genetic markers. Interestingly, the few primary glioblastomas with *IDH1* mutations also have a significantly better prognosis [5, 13-16].

The aim of this study was to validate the frequency of *IDH1* mutation in gliomas in the Mosul city and to correlate the IHD1 positivity with the type and grades of gliomas, and with age and sex of the patients.

## Material and Methods

This is a retro- and prospective case series study. In a period extended between 2008 and 2014, all types of intracranial gliomas of both sex and all age groups in the Mosul city were included in this study. Study carried out in Mosul Private Laboratory and in Al-Jamboree Teaching Hospital. The biopsies were processed histopathologically and paraffin-embedded blocks were sectioned on 4 micron thickness. Tumors proved to be gliomas were taken and were classified and graded according to last WHO Classification of the Central Nervous System Tumors [1]. Hereupon, 109 biopsies of adult, male and female, and pediatrics intracranial gliomas were collected with their clinical data including age and sex, MRI findings of site and side of affection and the provisional clinical diagnosis.

Ethical Approval was obtained from both Health Office and Medical College Ethical Review Committees.

### Immunohistochemical technique

Four micron thickness slides were deparaffinized and rehydrated. Antigen retrieval was carried out by autoclaving at 95-99 °C, for 20 minutes using retrieval solution (citrate puffer 10 mmol/L, pH 6.0). Sections then allowed cooling to a room temperature, followed by washing 3 times, each for 3 minutes, in phosphate buffered saline (PBS). Endogenous peroxidase activity was blocked by dipping sections in 3% hydrogen peroxidase blocker (Dako) for 10 minutes and washed in 3 changes of PBS. Sections were incubated with 1:20 diluted primary antibodies anti-human *IDH1* R132H (Dianova, GmbH, Hamburg, Germany, Mouse Monoclonal Antibody Clone H09) for 60 minutes, followed by washing twice for 3 minutes changes of PBS. Detection system using 2-steps polymer of HRP MR-2C, Polymer Detection Kit (Dianova Anti-Mouse, Rabbit, Universal Ms/Rb, PHA-70844) applied for 35 minutes for each step. Sections were washed twice by PBS and visualized using 3,3-diaminobenzidine (DAB) for 5-10 minutes. Finally, the sections were lightly counterstained with hematoxylin, dehydrated and mounted. Negative control sections were treated in the same way, but by the substitution of primary antibody with PBS. Positive control sections were taken from positive cases and were performed in each batch of staining.

Positive result show strong cytoplasmic staining which appears only in the tumor cells. Expression of *IDH1* was determined by visual semiquantitative assessment of the proportion of the positively stained tumor cells. Cases with ≥10% cells as positive, and cases with <10% cells were rated as negative [2, 13].

### Statistic analysis

Data were interpreted in form of frequencies and percentage. A chi square (χ^2^) test was used to associate the *IDH1* status and different study variables. Statistical significance was achieved when the p-value was less than or equal to 0.05.

Statistic analysis were performed using computer program Microsoft Excel Window 7 (Microsoft Corporation, NY, USA) and SPSS statistic program (SPSS Inc, Chicago, IL, USA).

## Results

### Clinical findings

**Figure 1:**
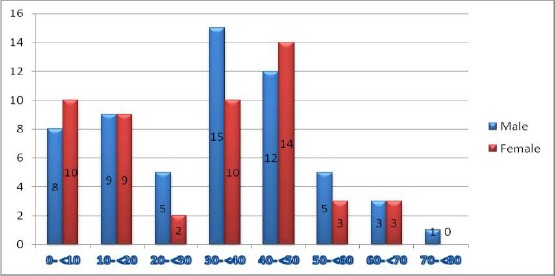
*Age and sex distribution of the intracranial gliomas*.

In a period of 5 years, 109 cases of intracranial gliomas were collected. The patients’ age range from 1.5 to 73 years with a mean age of 31.19 ± 15.36 years and a median of 32 years, most of the patients were in the third and fourth decades. There were 31 (28.44%) pediatric patients and 77 (71.55%) adults. Fifty eight (53.21%) were males and 51 (46.78%) were females and the male to female ratio was 1.13:1 (Figure 1).

### Histopathological findings

Astrocytic tumors were the predominant types of glioma and those were 34 (31.19%) primary glioblastomas, 9 (8.25%) secondary glioblastoma, 19 (17.43%) anaplastic astrocytomas, 16 (14.67%) low-grade diffused fibrillary and gemistocytic astrocytomas, 7 (6.42%) pilocytic astrocytomas and 1 (0.91%) subependymal giant cell astrocytoma, Whereas only 6 (5.50%) cases were oligodendro-gliomas/oligoastrocytomas. Twelve (11.0%) were conventional ependymomas, 3 (2.75%) were anaplastic ependymomas. There was 1 (0.91%) desmoplastic infantile ganglioglioma, and 1 (0.91%) ganglioglioma.

### Grading System

Tumors were graded according to the criteria established by WHO 2007 [1]. There were 10 (9.17%) cases grade I, 33 (30.27%) cases grade II, 23 (21.1%) cases grade III and the predominant grade was grade IV which was present in 43 (39.44%) cases as primary and secondary glioblastomas (Table 1).

**Table 1:** The Grades and Types of Gliomas.

Grade of Gliomas	No of cases	Type of Gliomas	Total	

			No	%
Grade-I	7	Pilocytic astrocytoma	10 (9.17%)	

	1	Subependymal giant cell astrocytoma		

	1	Desmoplastic infantile ganglioglioma		

	1	Ganglioglioma		

Grade-II	16	Low-grade diffuse astrocytoma	33 (30.27%)	

	5	Oligodendroglioma/oligoastrocytoma		

	12	Ependymoma		

Grade-III	19	Anaplastic astrocytoma	23 (21.1%)	

	1	Anaplastic oligodendroglioma		

	3	Anaplastic ependymoma		

Grade-IV	34	Primary glioblastoma	43 (39.44%)	

	9	Secondary glioblastoma		

Total	109		109 (100.0%)	

Gliomas were predominantly supratentorial in 80 (73.39%) cases, mainly on the right hemisphere especially in temproparietal lobe and commonly in adult patients. In contrary 29 (26.60%) gliomas were infratentorial commonly in the 4^th^ ventricle, majority were ependymomas and pilocytic astrocytomas, and seen mostly in children.

### *IDH1* and gliomas

The 10% cells positivity were the cornerstone point of *IDH1* mutation [2, 13]. Positive *IDH1* staining was observed in 38 (34.86%) cases of glioma. The secondary glioblastoma and the low-grade diffuse astrocytoma represent the largest groups of *IDH1* positivity, followed by oligoastrocytoma/oligo-dendroglioma in 50.0% of cases, and anaplastic astrocytoma in 47.36%. However, the p-value between the frequency of different types of gliomas ad the *IDH1* positivity failed to reach a statistical significance value (p-value = 0.056) (Table 2). *IDH1* was evenly expressed in both sexes,

**Table 2:** *IDH1* Status and the Types of Gliomas.

Types of Glioma		Total No	*IDH1* positivity No (%)	*IDH1* Negativity No (%)	P-value
Diffuse astrocytoma		16	10 (62.5%)	6 (37.5%)	0.056*

Anaplastic astrocytoma		19	9 (47.36%)	10 (52.63%)	

**Glioblastoma**	Primary glioblastoma	34	6 (17.64%)	28 (82.35%)	

	Secondary glioblastoma	9	8 (88.88%)	1 (11.11%)	

Oligodendroglioma/oligoastrocytoma		6	3 (50.0%)	3 (50.0%)	

Pilocytic astrocytoma		7	1 (14.28%)	6 (85.71%)	

Ependymoma		15	1 (6.66%)	14 (93.33%)	

Subependymal giant cell astrocytoma		1	0 (0.0%)	1 (100.0%)	

Desmoplastic infantile ganglioglioma		1	0 (0.00%)	1 (100.0%)	

Ganglioglioma		1	0 (0.00%)	1 (100.0%)	

Total		109	38 (34.86%)	71 (65.13%)	

* Chi squared was used.

Concerning the grades of gliomas, no significant relationship was identified between the *IDH1* positivity and the different grades of the tumors (Table 3).

**Table 3:** Correlation of *IDH1* Positivity and the Grades of Gliomas.

Grade of Gliomas	Total No case with *IDH1* Positivity	No of cases with *IDH1* Positivity	Type of Gliomas
Grade-I	1	1	Pilocytic astrocytoma

Grade-II	12	10	Low-grade diffuse astrocytoma

		2	Oligodendroglioma/Oligoastrocytoma

Grade-III	11	9	Anaplastic astrocytoma

		1	Anaplastic oligodendroglioma

		1	Anaplastic ependymoma

Grade-IV	14	6	Primary glioblastoma

		8	Secondary glioblastoma

Total	38	38	

Regarding the variants of glioblastoma, 4 were giant cell type, 1 of which was *IDH1* positive positivity for *IDH1*. Two were gliosarcomas, both were positive; also the 2 glioblastomas with primitive neuroectodermal tumor (PNET) components were positive for *IDH1* with granular rather than diffuse cytoplasmic staining. Lastly 1 out of 2 glioblastomas with oligodendroglioma component showed *IDH1* cytoplasmic positivity.

In regarding pediatric gliomas, apart from a single recurrent pilocytic astrocytoma and a primary glioblastoma which were *IDH1* positive, all others were *IDH1* negative. No oligodendrogliomas/oligo-astrocytomas or gangliogliomas were encountered below 15 years of age. Difference in age distribution of *IDH1* positivity between adult and pediatric gliomas is statistically highly significant (p<0.001) (Table 4).

**Table 4:** Distribution of *IDH1* Reactivity and the Age-related Gliomas.

Age-related Gliomas	Total	*IDH1*	Positivity	*IDH1*	Negativity	P-value*

	No	No.	%	No.	%	
Adult-related	78	36	(46.15%)	42	(53.84%)	

Pediatric-related	31	2	(6.45)	29	(93.54)	<0.001

Total	109	38	(34.86)	67	(61.46)	

* Chi squared was used.

## Discussion

*IDH1* mutation has became as a main diagnostic and prognostic biomarker for gliomas [13, 17]. *IDH1* mutations occurs in a vast majority of diffuse astrocytomas, oligodendrogliomas, and mixed oligoastrocytomas of WHO grades II and III and an earlier important findings in a fraction of secondary and primary glioblastomas [13,14,17,18].

**Figure 2:**
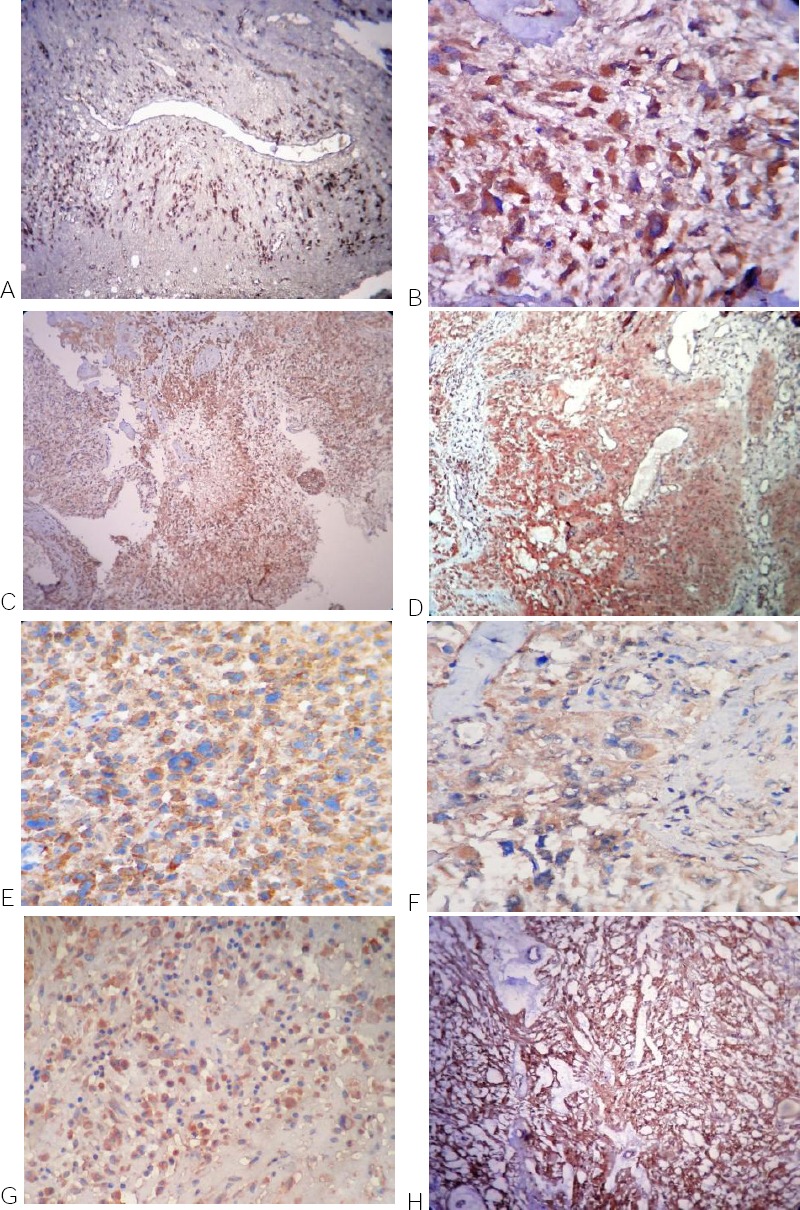
*A- Low-grade diffuse astrocytoma (IDH1 x100). B- Anaplastic astrocytoma (IDH1 x400). C- Glioblastoma (IDH1 x100). D- Gliosarcoma (IDH1 x400). E- Glioblastoma with PNET-like component (IDH1 x400). F- Glioblastoma with Oligodendroglioma component (IDH1 x400). G- Oligodendroglioma (IDH1 x100). H- Pilocytic astrocytoma (IDH1 x100)*.

The present work was the first study in Iraq to assess the immunohistochemical status of *IDH1* mutant in various types and grades of gliomas. In line with many previous studies [3-5,12,15,19-22], that reported a higher frequency of *IDH1* mutations in grade II gliomas compared with grades III and primary glioblastoma, the current showed highest *IDH1* mutations among low-grade diffused astrocytomas (62.5%), and in secondary glioblastoma (88.88%), between other grades and types of gliomas. However, in contrast to majority of the previous studies [2, 3, 5,18, 19, 23-27], the present study clarified a relatively higher degree of expression of the *IDH1* in primary glioblastoma (17.64%). This may be due to vague presentation, delay diagnosis and treatment of some of low grade gliomas that presented initially as primary glioblastomas. Nobusawa proposed that these primary glioblastomas with *IDH1* mutation actually represent secondary glioblastomas with an unusually short clinical presentation [15], (Table 5).

**Table 5:** The positivity of *IDH1* in primary glioblastomas in different studies.

Study	Region	Year	No. of cases	IDH1 positivity
Current study	Iraq	2014	109	17.64%
Leibetseder [23]	Austria	2013	70	39.30%
Takano et al [19]	Japan	2012	164	7.30%
Pollack et al [24]	USA	2011	106	16.30%
Toedt et al [25]	Germany	2011	131	8.00%
Jha et al [26]	India	2011	100	4.40%
Labussiere et al [27]	France	2010	1392	6.00%
Capper et al [5]	Germany	2009	345	4.00%
Yan et al [18]	U.K	2009	445	4.87%
Ichimura et al [3]	Sweden	2009	305	3.00%
Balss et al [2]	Germany	2008	685	7.00%

The presence of *IDH1* mutation in the majority of low grade astrocytomas confirms the neoplastic nature of the lesion and helps to differentiate the lesser cellular infiltrative tumor and /or tumor margin from gliosis particularly in a stereotactic biopsy [10, 14, 28, 29]. In the current study *IDH1* positivity was helpful in confirming the clinical diagnosis of the neoplastic nature of the lesion.

Concerning the 2 glioblastomas variant with PNET component, both were *IDH1* positive, which suggest the possibility of secondary glioblastoma originated from this tumor or the different histogeneic origin of this tumor from the primary Glioblastoma. In contrast other similar study showed reactivity of *IDH1* in a minority of glioblastoma with PNET component and argue against the sole of secondary glioblastoma [30]. Therefore, large-scale studies are necessary to conclude the facts.

In contrary to adult gliomas, pediatric low and high-grade gliomas did not express IDHI. There was only one pediatric primary glioblastoma and a recurrent case of pilocytic astrocytoma which expressed the *IDH1* mutation. This is in agreement with other related studies [2, 4, 14, 24, 31-33], which concluded no role of *IDH1*mutation in pediatric gliomas. This can be explained by the frequency, pathological spectrum and the anatomical location of gliomas in this age group. So this may highlight the differences in the pathogenesis between pediatrics and adult gliomas.

The prognostic role of *IDH1* in gliomas: *IDH1* mutation demonstrated by many studies as associated with prolonged survival. Furthermore, patients with IDH mutant glioblastomas showed longer survival than patients with glioblastomas, or even anaplastic astrocytomas, without IDH mutations [18, 21, 22, 34, 35]

In conclusion, *IDH1* mutation is commonly present in adult gliomas particularly low-grade gliomas, and secondary glioblastoma, with no sex predilection, but it has no role in pediatric gliomas.
